# Forecasting high-risk areas for dengue outbreaks in China: A trend analysis of *Aedes albopictus* and *Aedes aegypti* distributions from 2014 to 2030

**DOI:** 10.1371/journal.pntd.0013237

**Published:** 2025-07-09

**Authors:** Yulun Xie, Bingbing Wang, Qi Chen, Hongjie Wei, Yanshu Ke, Fang Xie, Xuhua Guan, Jia Rui, Tianmu Chen

**Affiliations:** 1 State Key Laboratory of Vaccines for Infectious Diseases, Xiang An Biomedicine Laboratory, National Innovation Platform for Industry-Education Integration in Vaccine Research, School of Public Health, Xiamen University, Xiamen, People’s Republic of China; 2 Hubei Provincial Center for Disease Control and Prevention, Wuhan, China; Basque Center for Applied Mathematics: Centro Vasco de Matematicas Aplicadas, SPAIN

## Abstract

**Background:**

Global warming, urbanization and the resumption of global population movements post-COVID-19 have made the prevention and control of dengue and its vector transmission more challenging. To tackle this issue, this study evaluated and predicted the suitable habitats of *Aedes albopictus* and *Aedes aegypti* in China. This study aims to identify the key influencing factors, analyze patterns of habitat expansion and contraction, and explore regions in China at risk of dengue fever outbreaks in the future.

**Methodology/principal findings:**

This study utilized mosquito distribution data from 2010 to 2023 and employed Maxent to map the distribution of suitable habitats. The key influencing factors and response curves were further analysed, and patterns of habitat expansion and contraction were investigated. The findings reveal that the main variables affecting the distributions of *Ae. albopictus* and *Ae. aegypti* are annual precipitation, annual mean temperature, and land use and land cover changes. The suitable habitat for *Ae. albopictus* shows a significant northward expansion trend, reaching large areas in Shandong and Henan province by 2030. The suitable habitat area for *Ae. albopictus* is increasing annually and can reach approximately 2.38 million square kilometers by 2030. Compared to the outbreak year of dengue fever in China in 2019, the suitable habitat area for *Ae. albopictus* in 2030 will increase by approximately 17.06%, with a growth of 2.57% in the sum of high-risk and medium-risk suitable habitat areas. In contrast, the suitable habitats of *Ae. aegypti* are primarily concentrated in Guangdong, Hainan and Yunnan Provinces.

**Conclusion/significance:**

This study compared the potential changes in the distributions of suitable habitats for *Ae. albopictus* and *Ae. aegypti* in 2014, 2019, and 2023 and predicted suitable habitats for 2030, as well as contraction and expansion trends in the suitable habitats of *Ae. albopictus*. The findings aim to identify regions at risk of future dengue fever outbreaks in China, providing a scientific basis for public health authorities to develop effective dengue prevention and control strategies.

## Author summary

Dengue fever is one of the most significant mosquito-borne diseases in tropical and subtropical regions. In June 2024, the World Health Organization (WHO) Pathogens Prioritization framework identified Orthoflavivirus denguei as a priority pathogen in all six WHO regions. In 2014 and 2019, China experienced severe dengue outbreaks. This study employs climate and environmental-economic variables in China in 2014, 2019, 2023 and 2030 to explore the main influencing factors of mosquito-borne transmission and the trend of habitat expansion and contraction, with the goal of assessing the risk areas for future dengue outbreaks in China. Research shows that the main factors that influence the distributions of *Ae. albopictus* and *Ae. aegypti* are the annual mean temperature, annual precipitation and urban/industrial/residential land. *Aedes albopictus* is the main vector of dengue fever transmission in China, and its suitable habitat will expand to the north and be widely distributed in Shandong Province by 2030. The suitable habitats of *Ae. aegypti* were mainly concentrated in Guangdong, Hainan and Yunnan Provinces. The results of this study provide a scientific basis and early warning system for identifying and predicting potential high-risk areas for dengue fever epidemics in China in the past 10 years and in the future in 2030.

## 1. Introduction

Dengue fever is one of the most significant mosquito-borne diseases in tropical and subtropical regions. Dengue cases surged worldwide in 2023 and 2024 [1,2]. The World Health Organization (WHO) assigned the highest level of emergency (grade 3) to the global increase in dengue cases [3].

China experienced severe outbreaks of dengue fever in 2014 and 2019 [4], indicating the potential for large-scale transmission of dengue fever in the country. With respect to global change, urbanization has directly promoted the spread of mosquito vectors, and the recovery of global population mobility post the coronavirus disease 2019 (COVID-19) pandemic has sharply increased the risk of dengue fever import into China, presenting greater challenges for its prevention and control. Even more concerning is the expanding distribution of *Aedes albopictus*, the main vector of dengue fever in China. Currently, *Ae. albopictus* has been reported in provinces and autonomous regions such as Liaoning, Hebei, Shanxi, Shaanxi, and Sichuan [5]. Given the combination of mosquito vector expansion and increased risk of imported cases, along with the lack of widespread dengue fever vaccination, the situation for dengue prevention and control in China has become more complex and urgent.

In this context, controlling the transmission vectors of dengue fever, *Ae. albopictus* and *Aedes aegypti*, is crucial. However, owing to China’s vast territory, which spans three climate zones, and the significant differences in climate and terrain, mosquito monitoring is challenging. This makes vector control and dengue prevention particularly difficult. Therefore, comprehensively assessing and dynamically monitoring the distributions of *Ae. albopictus* and *Ae. aegypti* in China, and updating dengue vector distribution maps, have become important for improving dengue surveillance and early warning capabilities, as well as formulating targeted control strategies.

To address these problems, this study employs an ecological niche model and utilizes the latest climate data and environmental-economic related variables to evaluate the distributions of *Ae. albopictus* and *Ae. aegypti* in China. Given that 2014 and 2019 witnessed significant dengue fever outbreaks in China, Maxent models were built to map the suitable habitat distribution and potential trend changes in *Ae. albopictus* and *Ae. aegypti* in 2014, 2019, and 2023. Furthermore, the model was used to predict the suitable habitat distribution for these mosquitoes in 2030.

The objective of this study was to pinpoint potential high-risk areas for future dengue outbreaks. The findings will offer scientific insights to public health professionals, aiding in the development of targeted mosquito vector management and monitoring strategies. Additionally, these findings will provide scientific backing for public health departments to devise strategies aimed at preventing and controlling dengue. Ultimately, these efforts will ultimately guide the implementation of effective public health interventions to control the transmission of dengue fever.

## 2. Methods

**Fig 1:**
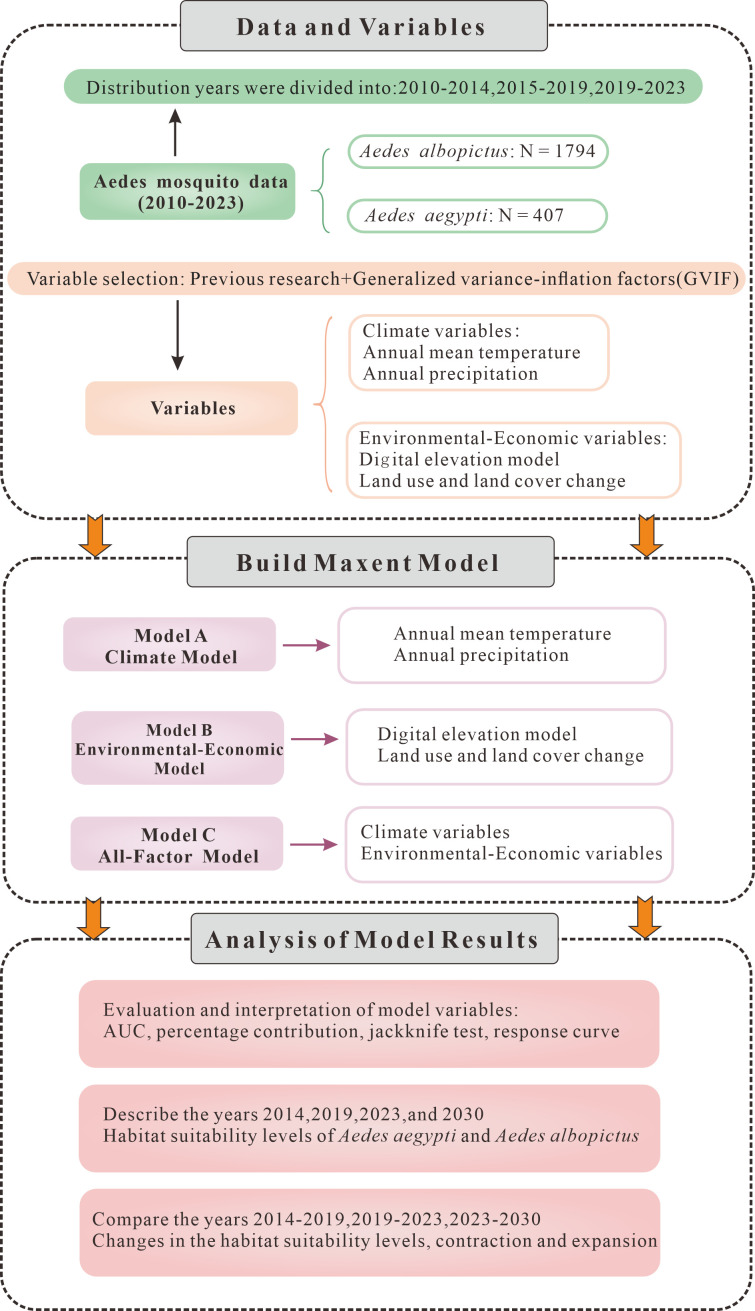
The technical flowchart.

The technical approach of this study is shown in Fig 1. In short, we divided the *Aedes* mosquito distribution data into three time periods and collected the corresponding climate variables and environmental-economic variables. Then, we constructed model A, model B, and model C based on different combinations of variables. Finally, we used AUC, response curves, and so on to evaluate and explain the role of the variables, and described the contraction and expansion of the suitable habitats in 2014, 2019, 2023, and 2030.

### 2.1. Data collection

#### 2.1.1. Mosquito data.

The occurrence data for *Ae. albopictus* and *Ae. aegypti* in this study were obtained from two primary sources. The first source is a comprehensive and systematic literature search of databases such as CNKI, SinoMed, and PubMed, which yielded mosquito coordinates from relevant literature published between 2010 and 2023. The second source is the Global Biodiversity Information Facility (GBIF) database (https://www.gbif.org/), which contains numerous global occurrence records of species. We filtered for *Ae. albopictus* and *Ae. aegypti* coordinate records in China from 2010 to 2023.

Considering the long-term trend changes in variables over time, species behavior changes, and the number of species points, we focused more on the distribution of newly added species points over five years for each study year. This approach better reflects the distribution trend changes of mosquitoes. We divided the mosquito coordinates into three time periods: 2010–2014, 2015–2019, and 2019–2023. There are 1,794 records of *Ae. albopictus* and 407 records of *Ae. aegypti*.

#### 2.1.2. Variable acquisition.

The climate variables include the annual mean temperature (AMT) and annual precipitation (APP) for the years 2014, 2019, 2023, and 2030. These data were obtained from the National Earth System Science Data Center (https://www.geodata.cn/index.html) [6–8]. For the future year of 2030, we selected the SSP245 scenario for the projections. The 2000 digital elevation model (DEM) was obtained from the Resource and Environmental Science Data Platform (RESDC: https://www.resdc.cn/). Land use and land cover change (LUCC) data for 2013, 2018, and 2023 were obtained from the Resource and Environmental Science Data Platform (RESDC: https://www.resdc.cn/doi:10.12078/2018070201). Since LUCC data for 2014 and 2019 were not included in this dataset and considering the small changes in LUCC over the years, we used LUCC data from the previous years, 2013 and 2018, as substitutes to ensure the continuity and reliability of the analysis. LUCC data for 2030 were obtained from the Global Change Research Data Publishing & Repository (https://geodoi.ac.cn/WebCn/Default.aspx) [9], also under the SSP245 scenario. LUCC data result from the combined effects of natural conditions and human activities (e.g., urbanization of residential areas). As the only categorical variable, we reclassified LUCC into six categories (arable land, forestland, grassland, urban/industrial/residential land, unused land, and water bodies) to standardize the values. These categories represent the different land use types and coverage changes in China. The map was obtained from GaryBikini (https://zenodo.org/records/10624971) [10].

All variables were clipped and adjusted to the geographical region of China via ArcGIS 10.8 and then resampled to a uniform resolution of 1 km × 1 km.

### 2.2. Data analysis

#### 2.2.1. Establishment of the Maxent model.

Maxent 3.4.3 (http://biodiversityinformatics.amnh.org) is widely used to assess and predict suitable habitat distributions of species because of its inherent stability and high accuracy [11]. Among various modelling methods, the Maxent model has demonstrated outstanding predictive performance in species niche modelling [12]. In short, the model uses the Maximum Entropy method to assess the probability of species occurrence. By using the known distribution locations of species and corresponding explanatory variables, the Maxent model analyzes the ecological requirements of the species, mapping them to different geographical areas to predict the potential distribution of the species in a specific region. For a more detailed understanding of the model’s principles and usage, please refer to its official website (http://biodiversityinformatics.amnh.org) and articles [13–15].

To differentiate and assess the impacts of various types of variables and their interactions on the prediction of habitat suitability, we constructed three models based on the original variables: climate model A, which included climate variables (AMT and APP); environmental-economic model B, which included LUCC and DEM variables; and all-factor model C, which included all the variables (Table 1).

**Table 1:** Model construction and variables.

Model	Variable type	Variables	Number of variables
A	Climate variables		2
		AMT	
		APP	
B	Environmental-Economic variables		2
		LUCC	
		DEM	
C	All-Factor variables		4
		AMT	
		APP	
		LUCC	
		DEM	

In this study, 75% of the species distribution points were set as training points, and the remaining 25% were set as testing points (randomly selected by the program). Each model was repeated 10 times, and the average value was taken as the final prediction result.

#### 2.2.2. Variables analysis.

Generalized variance-inflation factors (GVIFs) are calculated with *vif* function in *car* R package, with GVIF greater than 10 considered highly collinearity [16]. No variables exhibited high collinearity (S3 File).

The jackknife test reflects the effect of sequentially excluding variables and using variables alone [17]. The larger the gain obtained when only a single variable is used, the more information that a variable contains that is not present in other variables [18], which contributes more to the model’s predictive power.

#### 2.2.3. Model evaluation and division of habitat suitability.

We imported the variables into the Maxent model according to the intended model structure. The area under the receiver operating characteristic curve (AUC) was used to assess the predictive performance of the model. The larger the AUC value is, the better the model’s predictive ability: excellent (0.90–1.00), very good (0.80–0.90), good (0.70–0.80), fair (0.60–0.70), and poor (0.50–0.60) [19].

Considering both the model AUC values and the distribution and quantity of species occurrence points, we used the *Aedes* mosquito distribution data from 2015 to 2019 along with relevant variables to train the model, which was then used to predict the habitat suitability for *Aedes* mosquitoes in 2030. The suitability maps for *Ae. albopictus* and *Ae. aegypti* were generated in ArcGIS 10.8, where values ranged from 0 to 1, with higher values indicating greater suitability for mosquitoes. To more intuitively display the regional suitability risk, we categorized the habitat suitability values into four risk levels: 0.00–0.05 for uninhabitable habitat, 0.05–0.25 for low suitability habitat, 0.25–0.50 for medium suitability habitat, and 0.50–1.00 for high suitability habitat.

## 3. Results

### 3.1. Distribution

**Fig 2:**
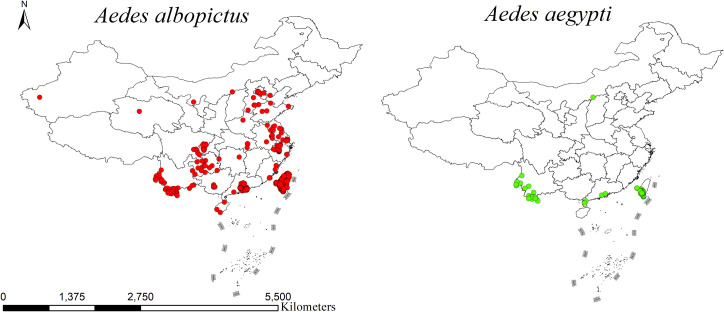
Distribution of *Aedes albopictus* and *Aedes aegypti* in China from 2010 to 2023. The map was obtained from GaryBikini (https://zenodo.org/records/10624971).

Using ArcGIS 10.8, the distribution points of *Ae. albopictus* and *Ae. aegypti* were visualized and displayed on a map of China, as shown in Fig 2. The results indicate that *Ae. albopictus* has a relatively wide distribution and is found in southern, eastern, and central China, with some scattered presence in the northern regions. In contrast, the distribution of *Ae. aegypti* in China is limited and sparse and is concentrated mainly in Guangdong, Yunnan, and Taiwan.

### 3.2. Model performance evaluation

In species distribution modelling, the AUC value is a commonly used metric for comparing the performance of different models. After 10 repetitions, all the tested models demonstrated excellent (AUC > 0.9) predictive performance. The results are shown in S4 File, where all-factor Model C consistently had the highest AUC values across all years.

### 3.3. Evaluation of influencing factors

To fully consider the interactions between variables, we analysed the years with the highest AUC in all-factor Model C (2023 for *Ae. albopictus* and 2019 for *Ae. aegypti*).

**Fig 3:**
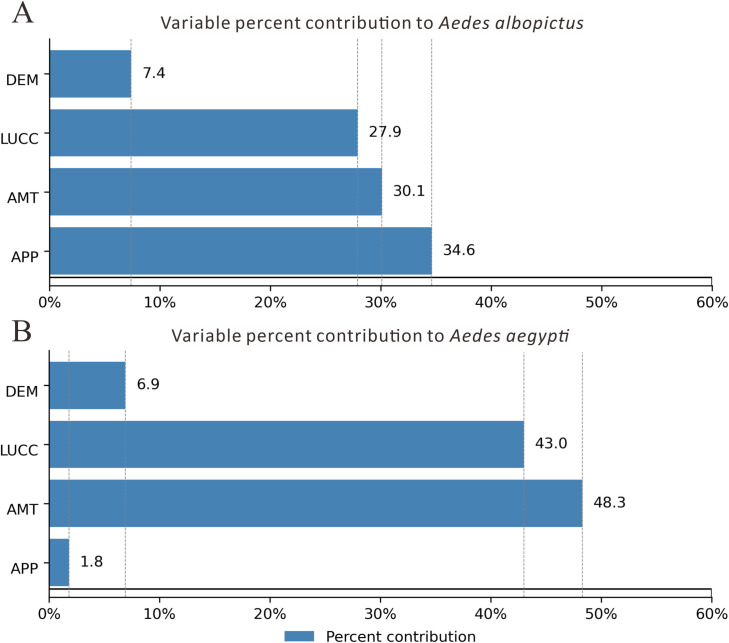
The average percentage contribution of variables across 10 runs. A. Variable percent contribution to *Aedes albopictus*. B. Variable percent contribution to *Aedes aegypti*.

The percentage contribution values indicate that, for *Ae. albopictus*, APP, AMT and LUCC contributed the most to improving model performance, accounting for more than 90% (92.6%) of the total. For *Ae. aegypti*, AMT and LUCC were the most significant contributors to improving model performance, accounting for more than 90% (91.3%) (Fig 3).

**Fig 4:**
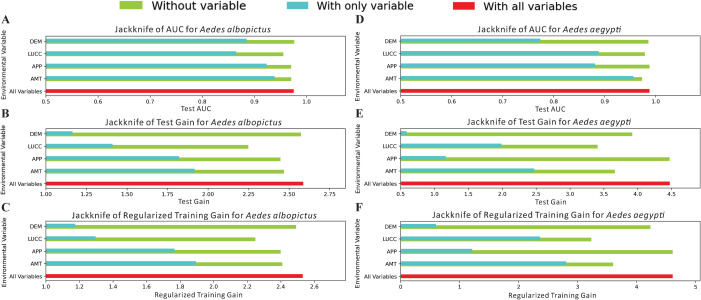
Jackknife test results. Panels A, B, and C represent the test AUC, test gain, and regularized training gain of *Aedes albopictus.* Panels D, E, and F represent the test AUC, test gain, and regularized training gain of *Aedes aegypti*. Light green indicates the impact on the model when this variable is excluded, and light blue indicates the independent impact of this variable to the model.

Considering all the jackknife test results (Fig 4), the impacts of the variables on *Ae. albopictus* and *Ae. aegypti* were consistent. When only a single variable was used for modelling, AMT, APP and LUCC had the greatest impact on the model’s predictive ability. Among these, the exclusion of LUCC had the most significant impact on the model’s overall performance.

Therefore, considering both the percentage contribution values and the jackknife test results, the main influencing variables for both *Ae. albopictus* and *Ae. aegypti* are APP, AMT and LUCC.

### 3.4. Response curve

**Fig 5:**
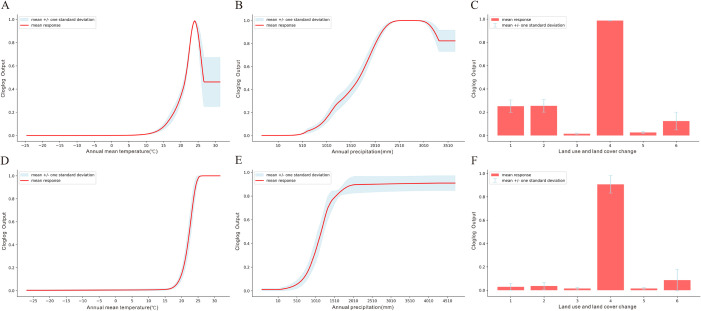
Response curve. Panels A, B and C show the AMT, APP, and LUCC response curves of *Aedes albopictus*. Panels D, E and F shows the AMT, APP, and LUCC response curves of *Aedes aegypti*. These curves show how each variable affects the Maxent prediction. The curves show how the predicted probability of presence changes. The red curves show the mean response of the 10 replicate Maxent runs, and the blue range represents the mean + /− one standard deviation.

Based on the Maxent output results and the reasonable range of variables, we generated response curves for the three most significant influencing variables (Fig 5). The mean + /- one standard deviation is an indicator that describes the central tendency and degree of dispersion of the results. *Aedes albopictus* is most active at around 25°C, with a minimum temperature threshold of 12.5°C (Fig 5A), while *Ae. aegypti* requires a relatively higher minimum temperature of around 17.5°C (Fig 5D). The predicted probability of both mosquito species increases with rising APP (Fig 5B and 5E). In terms of LUCC, urban/industrial/residential land had the greatest impact on the predicted probability of *Aedes* mosquitoes (Fig 5C and 5F).

### 3.5. Habitat suitability

On the basis of different variable combinations in models A, B and C, different models reflect the impact of considering AMT and APP, LUCC and DEM, as well as the comprehensive consideration of all factors affecting habitat suitability for *Aedes* mosquitoes.

**Fig 6:**
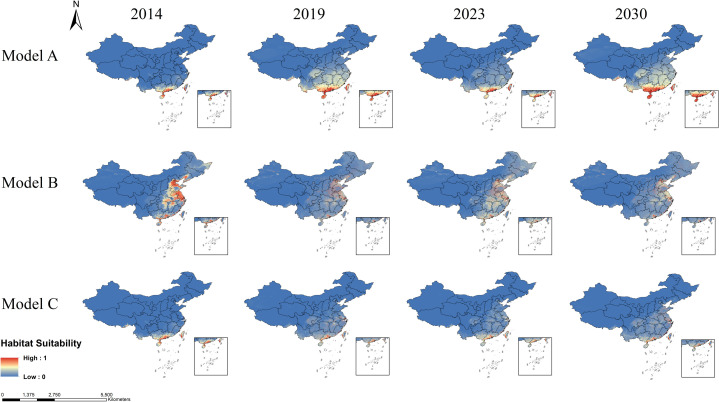
The suitable habitat range of *Aedes albopictus* across different years according to the three models. The habitat suitability values ranged from 0 to 1, with higher values indicating greater suitability for mosquitoes. The map was obtained from GaryBikini (https://zenodo.org/records/10624971).

**Fig 7:**
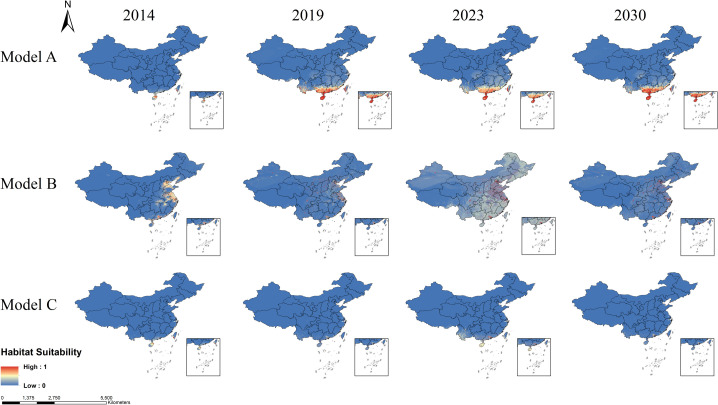
The suitable habitat range of *Aedes aegypti* across different years according to the three models. The habitat suitability values ranged from 0 to 1, with higher values indicating greater suitability for mosquitoes. The map was obtained from GaryBikini (https://zenodo.org/records/10624971).

Model A revealed that the suitable habitats for *Ae. albopictus* were concentrated in southern provinces, such as Guangdong, Hainan, Guangxi, and Taiwan, and gradually expanded northwards over time to regions such as Anhui, Jiangsu, and Shandong (Fig 6). The suitable habitats for *Ae. aegypti* are concentrated mainly in southern coastal provinces, including Guangdong, Guangxi, Hainan, and Taiwan (Fig 7). Model B, which considers LUCC and DEM variables, indicates that the suitable habitats for *Ae. albopictus* are primarily distributed in the eastern and southern provinces of China (Fig 6). The suitable habitats for *Ae. aegypti* are less stable, showing significant variation across years, but are also concentrated mainly in the eastern and southern provinces (Fig 7). Model C, which integrates AMT, APP, LUCC and DEM, revealed that the suitable habitats for *Ae. albopictus* gradually expanded from 2014 to 2030. Guangdong, Hainan, Guangxi, and Taiwan show consistently high suitability across all time points. Additionally, the suitable habitats have continuously expanded northwards over time, and by 2030, they have spread to most areas of Shandong and Henan (Fig 6). This model indicates that the suitable habitats for *Ae. aegypti* are relatively limited and are primarily concentrated in southern regions such as Guangdong, Hainan, and Yunnan (Fig 7).

The habitat suitability results (model C) indicate that the suitable habitats of *Ae. albopictus* are widely distributed in China, while the habitat suitability of *Ae. aegypti* are relatively limited, confined to a few areas in the south (Guangdong, Hainan, Yunnan, and Taiwan). Therefore, the following sections will mainly demonstrate the classification and assessment of the suitable habitats of *Ae. albopictus*, and the classification of the suitable habitat risk levels of *Ae. aegypti* is detailed in S1 Fig.

### 3.6. Classification of risk levels in terms of habitat suitability

**Fig 8:**
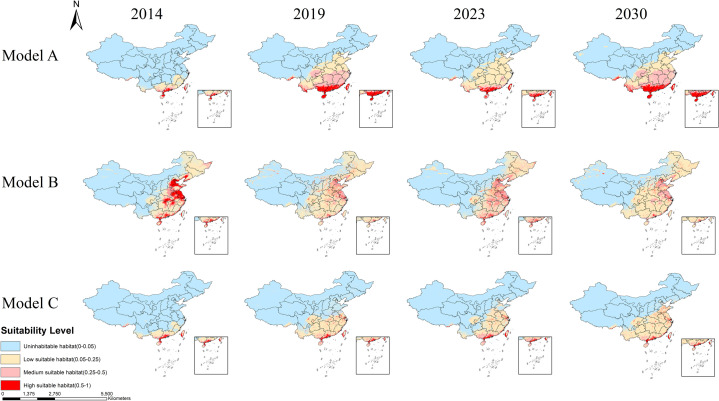
Distribution of habitat suitability classifications for *Aedes albopictus.* 0.00–0.05 indicates uninhabitable habitat, 0.05–0.25 indicates low suitable habitat, 0.25–0.50 indicates medium suitable habitat, and 0.50–1.00 indicates high suitable habitat. The map was obtained from GaryBikini (https://zenodo.org/records/10624971).

From a temporal perspective, all three models for *Ae. albopictus* show a continuous expansion of suitable habitat risk levels and ranges over time (Fig 8). In contrast, *Ae. aegypti* exhibited some fluctuations, with the risk levels and ranges being relatively consistent in 2014, 2019 and 2030 but expanding in 2023 (S1 Fig).

Compared with the results from the models (Fig 8), Model A, which considers climate variables, reveals a broader suitable habitat range with higher risk levels in central, southern, and eastern China and southwestern provinces such as Guizhou and Yunnan. Model B, which incorporates LUCC and DEM, predicts suitable habitats that are primarily distributed in eastern and southern China and even some parts of northeastern China. Model C, which integrates all the factors, reflects the comprehensive suitable habitat ranges for *Ae. albopictus*.

In terms of the distribution differences between the two species of mosquitoes (Figs 8, and S1), all three models for *Ae. albopictus* present relatively large suitable habitat ranges. In Model C, which integrates all the factors, the high-risk suitable habitats are primarily concentrated in Taiwan, Hainan, and Guangdong. However, some high-risk areas are gradually expanding northwards, reaching Shandong, Jiangsu, and Henan Provinces (Fig 8). In contrast, the suitable habitat range for *Ae. aegypti* in Model C was narrower, with high-risk suitable habitats scattered in parts of Hainan, Yunnan, and Guangdong (S1 Fig).

### 3.7. Expansion and contraction

**Fig 9:**
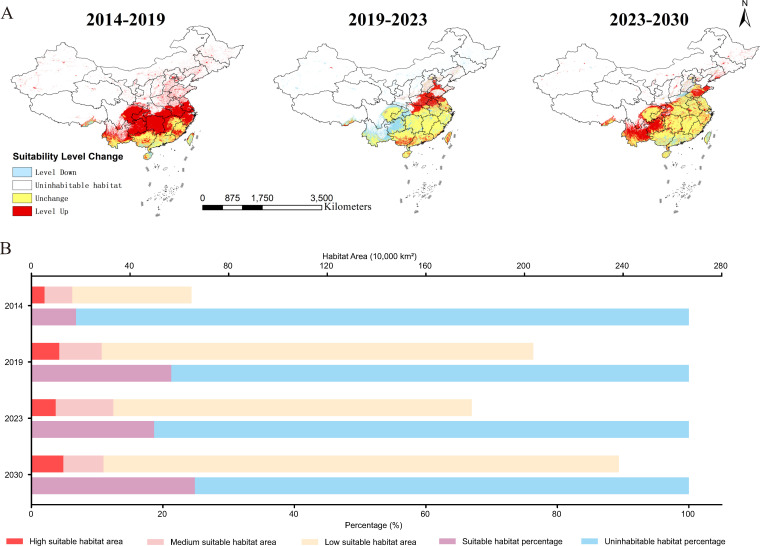
Changes in habitat suitability risk levels and the expansion and contraction of the habitat area of *Aedes albopictus.* A. The risk level changes, expansion and contraction of the suitable habitats for *Aedes albopictus* during the three time periods of 2014–2019, 2019–2023, and 2023–2030. B. The percentage of suitable and uninhabitable habitat areas for *Aedes albopictus* in each year, as well as the suitable habitat areas for each risk level (10,000 km^2^). The map was obtained from GaryBikini (https://zenodo.org/records/10624971).

In Model C, the changes in habitat suitability risk levels and the expansion and contraction of *Ae. albopictus* during 2014–2019, 2019–2023, and 2023–2030 are shown in Fig 9. The distribution of *Ae. albopictus* habitat suitability clearly tends to expand northwards, with large areas extending to Shandong by 2023 and 2030.

From a quantitative perspective, the suitable habitat area for *Ae. albopictus* is generally increasing annually and can reach approximately 2.38 million square kilometers by 2030. Compared to the outbreak year of dengue fever in China in 2019, the suitable habitat area for *Ae. albopictus* in 2030 will increase by approximately 17.06%, with a growth of 2.57% in the sum of high-risk and medium-risk suitable habitat areas (Fig 9).

## 4. Discussion

This study compared and predicted the changes in habitat suitability risk levels and ranges for the dengue fever vectors *Ae. albopictus* and *Ae. aegypti* in China for the years 2014, 2019, 2023, and 2030 to explore regions in China that may experience dengue outbreaks in the future.

From a temporal perspective, this study uses the latest climate and environmental-economic variables to assess the suitable habitats for *Aedes* mosquitoes, which is consistent with previous studies [20,21]. The high suitable habitats predicted for *Ae. albopictus*, such as Guangdong, Guangxi and Hainan, are regions in China with a high burden of dengue fever [4]. According to the habitat suitability results in 2014, the suitable habitats for *Aedes* mosquitoes were mainly concentrated in southern and southeastern China, which aligns with the outbreaks of dengue fever in Guangdong, Guangxi and other provinces in 2014 [22]. In 2019 [23], outbreaks occurred in Guangdong, Zhejiang, Jiangxi, and Hainan Provinces, and the outbreaks in Zhejiang and Jiangxi also corresponded with the predicted expansion and contraction of suitable habitats. Zhuowei Li [24] analysed the spatial and temporal distributions of dengue fever from 2019 to 2023 and reported that most of the southern regions of China are at risk of dengue transmission, with Southwest China and South China being key risk areas. This finding is also consistent with our 2019 and 2023 suitability results. In addition, cases of dengue fever in China have been reported in Shandong, Henan and Chongqing Provinces [25–27], which is also consistent with our prediction. According to the study by Zhao [28], there were cases of dengue fever in Shandong Province in 2023. On the basis of the results of suitable habitats for *Ae. albopictus* and the expansion and contraction of these areas, Shandong Province has gradually transitioned from having scattered suitable habitats to having large suitable habitats for *Ae. albopictus*, including some high-risk suitable habitats. The suitable habitat range for *Ae. albopictus* is generally increasing annually, and the high-risk and medium-risk habitat areas will gradually increase from 2014 to 2030. These results collectively indicate that the risk of dengue fever outbreaks can be reasonably predicted through the distribution of *Aedes* mosquitoes.

Comparing the years 2014, 2019, 2023, and 2030, the suitable habitats of *Ae. albopictus* will further expand northward in 2030. In addition to the southern regions such as Guangdong, Guangxi, Hainan, and Yunnan, which often report dengue fever cases, northern provinces like Shandong and Henan will also have large areas of new suitable habitats, and there will also be some high-risk level suitable habitats. The results of this study predict the areas in China where dengue fever may outbreak in the future through the analysis of the suitable habitats of *Aedes* mosquitoes, providing a reference for the prevention and control of dengue fever.

From the perspective of modelling, Model C, which takes into account all the factor variables, may provide a more comprehensive prediction of the suitable habitat range. However, it is worth noting that, through the aforementioned analysis of influencing factors, AMT and APP are two important factors affecting the distribution of suitable habitats for *Aedes* mosquitoes. Therefore, the results of Model A may also represent a warning that the addition of suitable habitat areas in Model A, compared with the results of Model C, may also lead to the spread of dengue fever cases in these areas. Notably, the prediction results for *Ae. aegypti* in Models B and C for 2023 show a relatively peculiar situation compared with other years. Model B and Model C in 2023 showed a greater expansion compared to other years for *Ae. aegypti*. However, taking into account the collected species distribution data and comparing it with other years, we tend to believe that this is a slightly biased result. This may be because, during the period from 2019 to 2023, there were fewer distribution points of *Ae. aegypti* species (only 24 records), and they were distributed across various types of land use and cover change (LUCC). Since both Model B and Model C take LUCC into account, coupled with the small number of species distribution points, the suitable habitat results for this period showed a significant expansion compared to other years. If we disregard the prediction results for *Ae. aegypti* in 2023, the prediction results for 2014, 2019, and 2030 all show a high degree of consistency in Model C (S1 Fig).

Analyze the differences between *Ae. albopictus* and *Ae. aegypti* from the perspective of suitable habitat. Some studies have shown that dengue fever outbreaks in China are mainly caused by *Ae. albopictus* [29,30], and this can also be seen from the results of the suitable habitat ranges of *Ae. albopictus* and *Ae. aegypti* in this study. The suitable habitat area for *Ae. albopictus* is generally increasing annually. Compared to the outbreak year of dengue fever in China in 2019, the suitable habitat area in 2030 will increase by approximately 17.06%, with a growth of 2.57% in the sum of high-risk and medium-risk suitable habitat areas. This may be due to the strong adaptability of *Ae. albopictus* to environmental and climate changes. Analysis of the response curves revealed that its minimum temperature requirement is approximately 5°C lower than that of *Ae. aegypti*, which may result in a broader distribution area for *Ae. albopictus*. In contrast, the suitable habitats of *Ae. aegypti* in China are relatively limited.

The analysis of influencing factors revealed that APP, AMT and LUCC are important factors affecting the habitat suitability of *Ae. albopictus* and *Ae. aegypti*. These findings are consistent with those of other studies. Mosquito density is closely related to local climate conditions [31], and high temperatures and precipitation each year promote mosquito activity [32]. Climate directly regulates mosquito density through temperature and precipitation [33]. Temperature not only affects the development rate, mortality, and adult lifespan of mosquitoes but also influences virus replication and transmission in vectors [34]. *Aedes* prefer to lay eggs and breed in water-filled containers, so changes in precipitation can affect mosquito density [35,36]. Recent studies have shown that winter temperatures have the most significant effect on the presence of *Ae. albopictus*, followed by summer precipitation [37]. Among these variables, excluding the LUCC variable has the most significant effect on model prediction according to the jackknife test. These findings suggest that LUCC might play a crucial role in providing complementary information for species survival and distribution. Even though the prediction performance of this variable alone may not be the highest, its role in the model is likely irreplaceable and may offer key insights that other variables cannot provide. The high-risk habitat areas for *Aedes* mosquitoes predicted in this study are located mainly in coastal cities and other economically developed regions. Urban/industrial/residential land has a much greater influence on the distribution of *Aedes* mosquitoes than other land use types do. Studies have shown that socioeconomic factors are related to immature populations of *Ae. albopictus* [38,39] and that population density and urbanization level can affect mosquito distribution [40].

This study makes attempts based on existing research. Unlike most similar studies that rely on WorldClim (https://www.worldclim.org) bioclimatic variables [41–44], which cover only large time spans (such as 1970–2000, 2021–2040, 2041–2060, 2061–2080, and 2081–2100), critical bioclimatic variables for the period from 2001 to 2020 are lacking. This limits the accurate assessment of recent trend changes, as well as comparisons with the dengue outbreak years in China (2014 and 2019). This study integrates the latest climate data from China and precisely selects relevant climate and environmental-economic variables for 2014, 2019, 2023, and 2030, analyzing the habitat suitability distributions of *Ae. albopictus* and *Ae. aegypti* at these time points. The variable data used in this study, for 2014, 2019, 2023, and 2030, not only reviews and analyzes the past outbreak years but also assesses the recent and future situations. By comparing with the outbreak years, it can provide risk alerts and warnings to deal with potential future dengue outbreaks. In addition, regarding the selection of species distribution point data, most studies [45–47] use long-term species records, while we focus on the species distribution points within each five-year period. This may reduce the impact of species distribution changes caused by long-term environmental changes, social changes, intervention measures, and other factors. At the same time, it may better capture the distribution changes of *Aedes* mosquitoes in recent years.

This study has certain limitations. First, GBIF and literature data may tend to record species distributions in urban areas, so these data may over-represent urban areas and under-represent natural or rural habitats. This uneven data distribution does indeed affect the reliability of the model and the accuracy of predictions. This leads the model to overestimate the distribution of *Aedes* mosquitoes in urban areas and underestimate the distribution in natural or rural habitats when making predictions. Although we have tried our best to conduct a comprehensive search of GBIF and literature data to achieve as complete a species record as possible, this objective limitation still exists. Second, due to the limited records of *Ae. aegypti* in certain periods, this may lead to an incomplete understanding of its actual distribution and habitat preferences. This incompleteness of the data may affect the accuracy of the model’s predictions of suitable habitats, and thus affect the reliability of the predicted trends. Third, the calculation of the suitable habitat area is influenced by factors such as coordinate system selection and resolution. The suitable habitat area provided in this study is not very accurate and is mainly used for predicting the trends of contraction and expansion. Future research should be dedicated to collecting more comprehensive and representative *Aedes* mosquito distribution data, or improving the methodology, in order to enhance the accuracy and reliability of predictions.

This study provides important scientific evidence for the development of vector-borne disease prevention and control strategies. This study not only has reference value for the prevention and control of dengue fever but also offers insights for the control of other vector-borne diseases. On the basis of the results of this study, monitoring *Ae. aegypti* and *Ae. albopictus* in regions with high habitat suitability risk levels, as identified by the models, and establishing effective monitoring and risk assessment programs are recommended. This will enable timely tracking of mosquito vector dynamics and further validate the predictive accuracy of the models. Additionally, the increased amount of monitoring data contributes to improving the precision and applicability of the models. Future research can build on this study to further develop seasonal dynamic models and incorporate more relevant variables, especially socioeconomic factors and human activities.

## 5. Conclusions

This study compared the potential changes in the distributions of suitable habitats for two mosquito species, *Ae. albopictus* and *Ae. Aegypti*, across the year 2014, 2019, and 2023. Furthermore, it predicted the suitable habitats for these species in the year 2030. The findings of the study unveil that the primary factors influencing the distributions of *Ae. albopictus* and *Ae. aegypti*, which are the main vectors of dengue fever in China, encompass annual precipitation, annual mean temperatures, as well as land use and land cover changes. Notably, the suitable habitat for *Ae. albopictus* demonstrates a prominent trend of expansion towards the north, anticipated to encompass vast regions in Shandong and Henan Province by 2030. The suitable habitat area for *Ae. albopictus* is generally increasing annually. When compared to the year 2019, which witnessed an outbreak of dengue fever in China, the suitable habitat area for *Ae. albopictus* in 2030 will increase by approximately 17.06%. Additionally, there will be a 2.57% growth in the sum of high-risk and medium-risk suitable habitat areas. In contrast, the suitable habitats of *Ae. aegypti* are primarily concentrated in Guangdong, Hainan and Yunnan Provinces. The findings of this study provide a scientific basis for vector-borne disease prevention and control, particularly in dengue fever control, and offer valuable information for identifying and predicting high-risk areas for potential future outbreaks of dengue fever, thereby promoting the precise implementation of public health policies.

## Supporting information

S1 File *Aedes albopictus* records from 2010 to 2023. (XLSX)

S2 File *Aedes aegypti* records from 2010 to 2023. (XLSX)

S3 File Generalized variance-inflation factors (GVIFs). (DOCX)

S4 File AUC values of each model. (DOCX)

S5 File The data of *Aedes aegypti* for calculating GVIF. (CSV)

S6 File The data of *Aedes albopictus* for calculating GVIF. (CSV)

S7 File *Aedes aegypti* Jackknife test result. (XLSX)

S8 File *Aedes albopictus* Jackknife test result. (XLSX)

S9 File Response curve data of *Aedes aegypti.* (XLSX)

S10 File Response curve data of *Aedes albopictus.* (XLSX)

S11 File Suitable habitat area. (XLSX)

S1 Fig Distribution of habitat suitability classifications for *Aedes aegypti.* 0.00–0.05 indicates uninhabitable habitat, 0.05–0.25 indicates low suitable habitat, 0.25–0.50 indicates medium suitable habitat, and 0.50–1.00 indicates high suitable habitat. The map was obtained from GaryBikini (https://zenodo.org/records/10624971).
(TIF)
