# Supplementary material for: Forecasting high-risk areas for dengue outbreaks in China: A trend analysis of Aedes albopictus and Aedes aegypti distributions from 2014 to 2030
Source: PLoS Negl Trop Dis. 2025 Jul 9;19(7):e0013237. doi: 10.1371/journal.pntd.0013237 (PMC12240387; doi:10.1371/journal.pntd.0013237)
Supplement: S3 File — (DOCX) [file pntd.0013237.s003.docx]

Considering the long-term trend changes in climate and other variables, as well as the range and number of species distribution points, we used AMT and APP for 2019, LUCC data for 2018, and the DEM for 2000 as the basis. Variable data were extracted according to the distribution of *Ae. albopictus* and *Ae. aegypti* species points from 2010-2023. Model A contains the annual mean temperature (AMT) and annual precipitation (APP). Model B includes land use and land cover change (LUCC) and digital elevation model (DEM). Model C contains the annual mean temperature (AMT), annual precipitation (APP), land use and land cover change (LUCC) and digital elevation model (DEM). Generalized variance-inflation factors(GVIFs) are calculated with *vif* function in *car* R package. During the calculation, we deleted the rows containing missing values (-9999).

1. *Aedes albopictus*

For model A, which contains only continuous variables, there is no need to perform the adjustment of GVIF^(1/(2*Df))^.

|  | Model A | | Model B | | Model C | | | |
| --- | --- | --- | --- | --- | --- | --- | --- | --- |
|  | AMT | APP | LUCC | DEM | AMT | APP | LUCC | DEM |
| GVIF | 1.22 | 1.22 | 1.12 | 1.12 | 2.00 | 1.49 | 1.37 | 1.65 |
| Df | 1 | 1 | 5 | 1 | 1 | 1 | 5 | 1 |
| GVIF^(1/(2*Df))^ | - | - | 1.01 | 1.06 | 1.41 | 1.22 | 1.03 | 1.29 |

*2.Aedes aegypti*

For model A, which contains only continuous variables, there is no need to perform the adjustment of GVIF^(1/(2*Df))^. In the distribution of *Aedes aegypti*, there is no "unused land" land use type, so the degrees of freedom (Df) is 4.

|  | Model A | | Model B | | Model C | | | |
| --- | --- | --- | --- | --- | --- | --- | --- | --- |
|  | AMT | APP | LUCC | DEM | AMT | APP | LUCC | DEM |
| GVIF | 1.13 | 1.13 | 1.05 | 1.05 | 2.89 | 1.35 | 1.24 | 3.12 |
| Df | 1 | 1 | 4 | 1 | 1 | 1 | 4 | 1 |
| GVIF^(1/(2*Df))^ | - | - | 1.01 | 1.02 | 1.70 | 1.16 | 1.03 | 1.77 |
