# Supplementary material for: Forecasting high-risk areas for dengue outbreaks in China: A trend analysis of Aedes albopictus and Aedes aegypti distributions from 2014 to 2030
Source: PLoS Negl Trop Dis. 2025 Jul 9;19(7):e0013237. doi: 10.1371/journal.pntd.0013237 (PMC12240387; doi:10.1371/journal.pntd.0013237)
Supplement: S4 File — (DOCX) [file pntd.0013237.s004.docx]

Table S4. AUC values of each model

|  | *Aedes albopictus* AUC | | | *Aedes aegypti* AUC | | |
| --- | --- | --- | --- | --- | --- | --- |
|  | 2014 | 2019 | 2023 | 2014 | 2019 | 2023 |
| Model A | 0.948 | 0.926 | 0.947 | 0.987 | 0.965 | 0.983 |
| Model B | 0.931 | 0.934 | 0.936 | 0.965 | 0.977 | 0.903 |
| Model C | 0.950 | 0.966 | 0.976 | 0.987 | 0.998 | 0.997 |

Model A contains the annual mean temperature (AMT) and annual precipitation (APP). Model B includes land use and land cover change (LUCC) and digital elevation model (DEM). Model C contains the annual mean temperature (AMT), annual precipitation (APP), land use and land cover change (LUCC) and digital elevation model (DEM). The values in the table are the AUC values.
