# Supplementary figures and images for: Forecasting high-risk areas for dengue outbreaks in China: A trend analysis of Aedes albopictus and Aedes aegypti distributions from 2014 to 2030
Source: PLoS Negl Trop Dis. 2025 Jul 9;19(7):e0013237. doi: 10.1371/journal.pntd.0013237 (PMC12240387; doi:10.1371/journal.pntd.0013237)

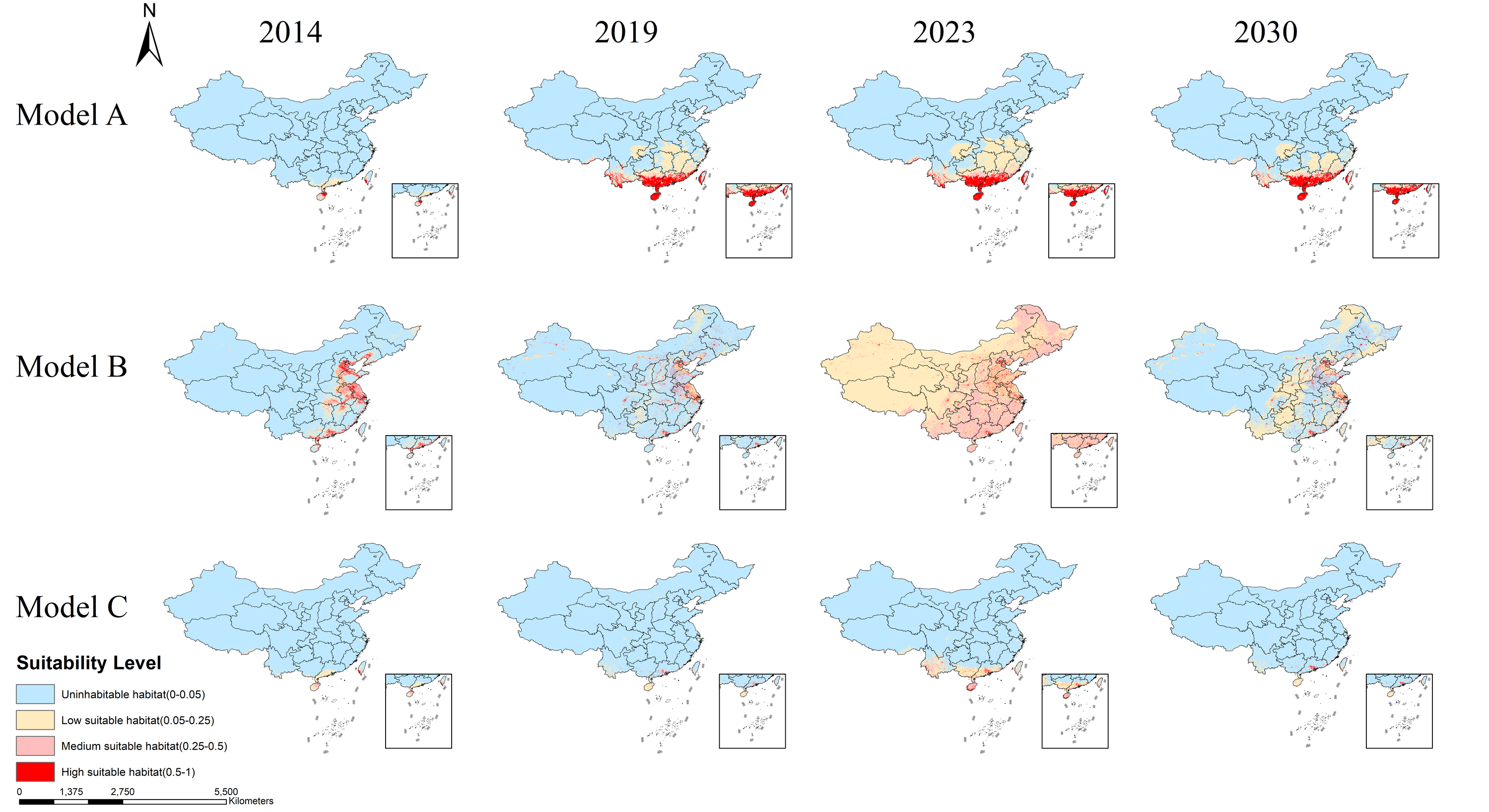

Supplement: S1 Fig — 0.00–0.05 indicates uninhabitable habitat, 0.05–0.25 indicates low suitable habitat, 0.25–0.50 indicates medium suitable habitat, and 0.50–1.00 indicates high suitable habitat. The map was obtained from GaryBikini (https://zenodo.org/records/10624971). (TIF) [file pntd.0013237.s012.tif]
